# Garland of Erythroblasts around a Macrophage: Erythroblastic Island

**DOI:** 10.4274/tjh.galenos.2019.2019.0398

**Published:** 2020-05-06

**Authors:** Chandan Kumar, Garima Jain, Anita Chopra

**Affiliations:** 1 Laboratory Oncology Unit, Dr. B.R.A.I.R.C.H., All India Institute of Medical Sciences, New Delhi, India

**Keywords:** Erythroblastic island, Macrophage

Erythroblastic islands (EBIs) were first described by Marcel Bessis in 1958 [1] as functional units of erythropoiesis where a central macrophage functions as a “nurse” cell [2].

We found a picturesque erythroblastic island with a central macrophage encircled by a garland of erythroid cells (Figure 1) in the bone marrow aspirate (BMA) of a 43-year-old man diagnosed with diffuse large B-cell lymphoma (DLBCL) on lymph node biopsy. BMA was done as a part of the staging workup, which was cellular and showed normoblastic erythroid hyperplasia and no evidence of lymphoma infiltration. Many macrophages surrounded by erythroid precursors (EBIs) were also seen.

Early descriptions of EBIs did not receive much attention because of their infrequent occurrence in BMA due to their distortion during smear preparation [2]. Some recent studies [3,4] have suggested that EBIs can boost the number of red blood cells produced in vivo during stress and can also act as potential targets in the treatment of inappropriately accelerated erythropoiesis in disease conditions like β-thalassemia and polycythemia vera. While progress is being made in understanding the role of the central macrophage of an EBI in regulating erythropoiesis, many questions remain unanswered, such as its precise physiology, clinical relevance, and contribution to the pathology of erythropoiesis in benign and malignant hematopoietic disorders.

## Figures and Tables

**Figure 1:**
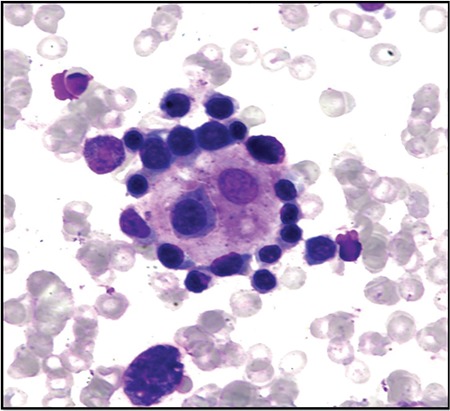
Bone marrow aspirate showing erythroid precursors surrounding a central macrophage: erythroblastic island (Jenner and Giemsa, 1000^x^).
